# Early Diagnostic Measures to Confirm the Diagnosis of Human Prion Diseases

**DOI:** 10.7759/cureus.39412

**Published:** 2023-05-23

**Authors:** Nisha Desai, Taylor Purzycki

**Affiliations:** 1 Internal Medicine, Overlook Medical Center, Summit, USA

**Keywords:** diagnose, general internal medicine, neurology and critical care, neurology, prion diseases

## Abstract

Human prion diseases are a group of rare and fatal diseases without a cure. Symptoms include rapidly progressive dementia, ataxia, myoclonus, akinetic mutism, and visual disturbances. A broad differential is required to consider prion disease as a diagnosis and rule out other conditions. Historically, to confirm the diagnosis of prion disease, a brain biopsy was needed. Over the past few decades, brain MRI, video electroencephalogram, lumbar puncture results, and a thorough clinical assessment have helped arrive at a probable diagnosis. We present the case of a 60-year-old female with a rapidly worsening altered mental status who received an early diagnosis of prion disease with the help of imaging and lab results. This case shows that a timely diagnosis of prion disease is important to allow the patient and their families to prepare for the inevitable fatality of the disease and discuss the goals of care.

## Introduction

Human prion diseases are a group of rare, transmissible, and fatal neurodegenerative diseases with a long incubation period, but a rapidly progressive clinical course. The incidence of prion disease is approximately 1.2 cases per million population annually [1]. The diagnosis is very difficult to make as it mimics several other conditions, such as autoimmune/antibody-mediated disorders, Alzheimer’s disease, frontotemporal dementia, vascular dementia, Lewy body dementia, encephalitis, multiple system atrophy, infection or neoplasms [2,3]. A definitive diagnosis of prion disease is made via brain biopsy or postmortem [2,3]. However, clinical judgment with imaging and test results can hint at the diagnosis. Unfortunately, the disease is fatal with no cure [1]. An early diagnosis can provide patients and their families time to understand the disease and decide on an appropriate goal of care for the patient, and avoid any unnecessary expensive investigations and treatments. We present a case report of a patient with rapidly deteriorating dementia in which a thorough clinical assessment, electroencephalogram, brain MRI, and lumbar puncture helped narrow down the differentials to arrive at the diagnosis.

## Case presentation

A 60-year-old Caucasian woman with bilateral lacunar ischemic infarcts in 2015 with a resolution of residual deficits, protein C deficiency, and depression presented with altered mental status and gait unsteadiness. Given her mentation, the history was obtained by her husband at the bedside. After the last stroke, the patient had a complete resolution of right-sided weakness and aphasia with aggressive therapy. She remained fully independent and worked full-time. Three months prior to presentation to the ED, the patient was laid off from her job and became more anxious and depressed. Over the last two months, the husband noticed a gradual deterioration in mental status and functionality, increased irritability, anxiety, and lethargy. The patient became poorly responsive to verbal stimuli and stared into space when spoken to, increasingly bed-bound, and fully dependent on her husband for her activities of daily living. She was taken to the ED where the brain MRI was initially read as unremarkable and was sent home. She now presented one month after her last ED visit with visual hallucinations, disorganized thinking, aggressiveness, gait unsteadiness, lack of coordination, and inability to perform her activities of daily living. She was initially admitted to the inpatient psychiatric unit, but despite medication optimization with selective serotonin reuptake inhibitors (SSRIs), antipsychotics, and benzodiazepines for a diagnosis of major depressive disorder with psychotic features, she continued to rapidly deteriorate. Due to her worsening altered mental status, the decision was made to transfer the patient to the medical service for further evaluation of a recurrent stroke versus other diseases causing rapid cognitive decline, such as prion.

On physical exam, the patient was alert and oriented to self only, and startled easily to light. Pupils were equal and reactive to light with limited extraocular movements. No facial asymmetry. Cranial nerves VII-VII were intact. We were unable to assess full motor and sensory along with a finger-to-nose test due to the patient’s inability to follow directions, but the patient was able to move both arms and legs symmetrically against gravity. Asterixis and myoclonus were present bilaterally. Deep tendon reflexes were absent throughout. Gait revealed a short shuffling gait.

**Figure 1:**
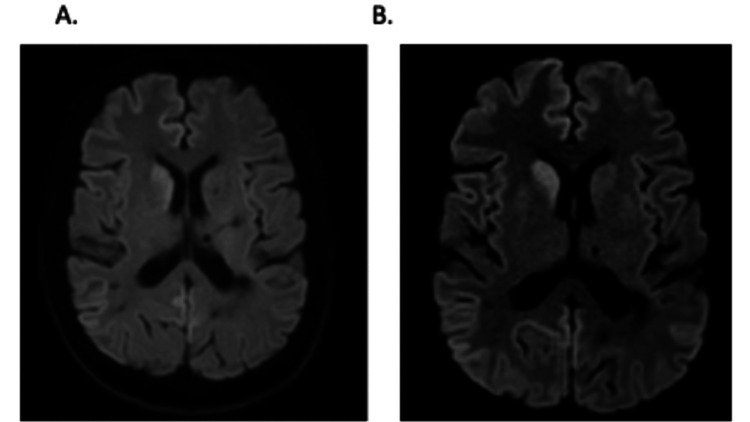
MRI of the patient's brain A: Initial brain MRI of the patient one month prior to presentation B: The post-admission diffusion-weighted image and T2-FLAIR brain MRI shows cortical diffusion abnormality asymmetrically involving the right cerebral hemisphere, basal ganglia diffusion abnormality with asymmetric involvement of the right caudate head and anterior lentiform nucleus, cortical ribboning, and confluent supratentorial and pontine white matter hyperintensity. FLAIR: Fluid-attenuated inversion recovery

As part of further workup, an MRI of the brain was repeated. Brain MRI (Figure 1B) showed a constellation of findings consistent with prion disease compared to the MRI one month prior (Figure 1A). The MRI of the brain now showed pronounced cortical diffusion abnormality asymmetrically involving the right cerebral hemisphere, basal ganglia diffusion abnormality with asymmetric involvement of the right caudate head and anterior lentiform nucleus, cortical ribboning, and confluent supratentorial and pontine white matter hyperintensity (Figure 1B). Traditionally, the MRI findings of bilateral high signals in the caudate and putamen with cortical ribboning are classic features of prion disease [4]. Complete blood counts, complete metabolic panel, thyroid stimulating hormone, ammonia, urine culture, vitamin B12, vitamin D, folic acid, Lyme disease testing, antinuclear antibodies, C-reactive protein, erythrocyte sedimentation rate, rheumatoid factor, and syphilis antibody were relatively unremarkable. Video encephalogram (VEEG) showed bilateral polymorphic periodic sharp and spike waves up to 2Hz with superimposed faster frequencies at 18 to 25Hz with no epileptiform activity. A lumbar puncture showed normal WBC, glucose, and protein with findings negative for xanthochromia, acid-fast bacillus, bacteria/fungal organisms, cryptococcus, Lyme, West Nile, Epstein Barr Virus, *Toxoplasma gondii*, JC virus, syphilis testing, oligoclonal banding, myelin basic protein, paraneoplastic autoantibodies, angiotensin-converting enzyme, and malignant cells. However, the real-time quaking-induced conversion (RT-QuIC) lab test returned after 10 days was positive, showing T-tau protein greater than 200,000 pg/ml (normal: 1-1149 pg/mL) and 14-3-3 gamma 116,000 AU/mL (normal <1999 AU/mL).

The diagnosis of prion disease was established via clinical assessment of the rapidly deteriorating dementia, MRI findings of bilateral high signal in the caudate with cortical ribboning, VEEG patterns of bilateral polymorphic periodic sharp and spike waves, and lumbar puncture results positive for RT-QuIC. The rapid course of the disease, poor prognosis, and lack of curative treatment was explained to the family. Palliative care was consulted, and the family decided to pursue hospice measures for the patient.

## Discussion

Human prion diseases are a group of rare and fatal neurodegenerative diseases caused by the misfolding of the normal cellular prion protein (PrPc) into an abnormal misfolded pathological form (PrPSc) [2]. The PrPSc configuration is resistant to proteases and therefore serves as a template for replication, leading to an accumulation of the abnormal prion protein in the brain [2]. This rapidly exponential rate of replication causes significant deterioration in mental status. The accumulation and aggregation of PrPSc in the brain lead to significant neurodegeneration, neuronal vacuolation, gliosis, and a spongiform appearance [2]. The most common human prion disease is sporadic Creutzfeldt-Jakob disease (sCJD) seen in 85% to 90% of the cases, but others include familial, variant, and iatrogenic forms. This condition is prevalent between the ages of 55 and 75 years and the eighth decade and seen in both genders equally [1]. The mortality rate from sCJD is approximately 1.2 cases per million people annually in the United States [1].

The clinical presentations of sCJD consist of various common symptoms such as language disturbances, neuropsychiatric behavioral changes, rapidly progressing dementia, cerebellar ataxia, myoclonus, pyramidal/extrapyramidal signs, visual disturbances, weakness, and possibly even hallucinations [1]. Similarly, our patient’s presentation shared symptoms of neuropsychiatric behavioral changes with an increase in agitation, deteriorating mental status, ataxia, visual disturbances, generalized weakness, and visual hallucinations. By the time patients present with these symptoms, they are unfortunately already in the latter part of the disease course. The progression of symptoms occurs rapidly, with significant impairment and even death within six months of disease onset. The differential diagnosis for rapidly progressive mental status change includes autoimmune diseases, infectious and metabolic encephalopathies, seizures, subdural hemorrhages, and neurodegenerative diseases, which must be considered. Due to the rapid progression of the disease, it is important to arrive at a diagnosis early on.

**Figure 2:**
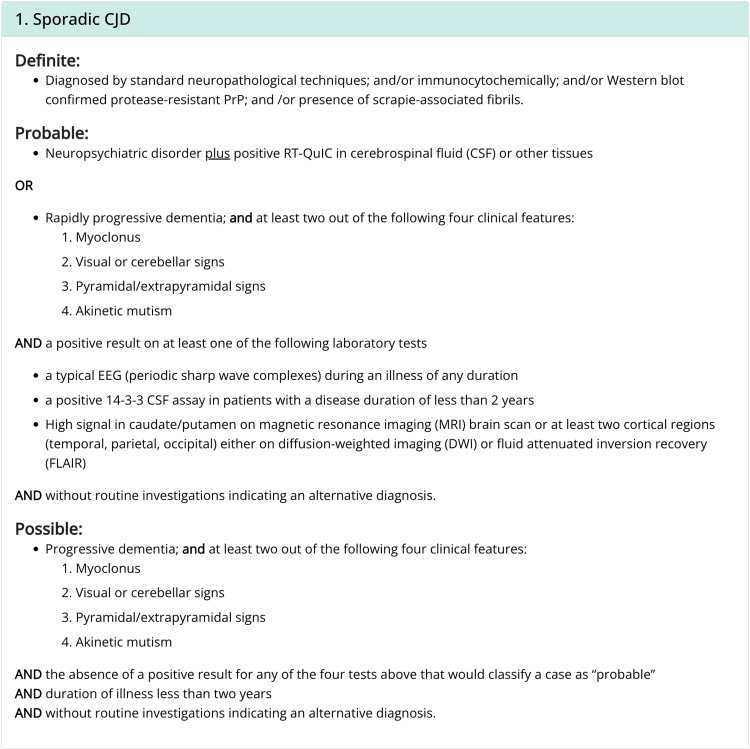
The CDC’s diagnostic criteria for sCJD, 2018 sCJD: Sporadic Creutzfeldt-Jakob Disease

In the past few decades, a diagnosis of prion disease has been made using clinical presentation in conjunction with imaging and lab studies; a brain biopsy is no longer needed for a confirmatory diagnosis. A multicenter international study provides a diagnostic criterion for sCJD aside from the definitive brain biopsy [5]. The CDC also provides methods to come up with a definite, probable, and/or possible diagnosis for sCJD (Figure 2) [6]. Diagnostic testing includes neuroimaging of the brain, VEEG, and lumbar puncture. Brain MRI with diffusion-weighted imaging (DWI) and T2-fluid-attenuated inversion recovery (FLAIR) with contrast may reveal a high signal in the caudate/putamen region on either DWI or FLAIR. A classic "hockey-stick" sign may be seen with the hyperintense signals bilaterally of the dorsomedial thalamic and pulvinar regions. The VEEG may show signs of typical periodic sharp wave complexes in the later course of the disease. Cerebrospinal fluid (CSF) analysis of tau protein and 14-3-3 protein can be helpful to support the diagnosis of sCJD but is nonspecific [1]. Recently, a new CSF analysis via the technique of RT-QuIC is now available to test the presence of misfolded prion proteins with a 77% to 89% sensitivity and 99% specificity [7]. An increase in tau and 14-3-3 proteins may be seen in 90% of patients with prion disease, with a poor specificity of 70%, as they can be found elevated in other settings such as acute ischemia, post-seizures, or inflammatory brain disease [8].

This case report describes a 60-year-old female with rapidly progressing dementia over the course of three months who was evaluated for a broad range of differentials, including psychiatric conditions, infectious etiology, stroke, and seizures. It is important for physicians to consider the diagnosis of prions as a differential even though it is very rare and only presents as 1.2 cases per million people annually worldwide [1]. Although a confirmative diagnosis is made via brain biopsy or postmortem, there are multiple other modalities for approaching the probable diagnosis of sCJD. A physician's clinical assessment of the rapidly deteriorating dementia, excluding other potential causes, MRI brain findings, CSF analysis positive for RT-QuIC, and VEEG showing periodic sharp wave complexes can confirm the diagnosis of sCJD.

Our patient’s clinical presentation and test results were suggestive of sCJD, and the disease progression was explained thoroughly to the family. It was difficult for the family to understand the lack of a cure and the near-future demise of the patient. The family had time to process this life-changing condition, understand the fated outcome, and plan for the patient’s future care by ensuring her comfort. It is important to reach a diagnosis of this rapidly deadly disease to provide the patient and their family with sufficient time to understand the disease and prepare for the inevitable fatality, as there is no cure for this disease.

## Conclusions

Human prion disease is a rare condition with a fatal outcome. It is important to arrive at an early diagnosis for this condition, as the disease has a rapid progression leading ultimately to death. The diagnosis is no longer exclusively via brain biopsy or postmortem autopsy. A constellation of findings on tests, such as MRI of the brain, positive RT-QuIc, and VEEG, can determine a probable diagnosis. Early diagnosis can avoid further unnecessary testing and treatment and can help physicians make appropriate plans for care with the patient and their family.
